# Advances in the Diagnosis and Management of Psychosis

**DOI:** 10.3390/diagnostics13091517

**Published:** 2023-04-23

**Authors:** Drozdstoy Stoyanov

**Affiliations:** 1 Department of Psychiatry and Medical Psychology, Research Institute, Medical University Plovdiv, 4002 Plovdiv, Bulgaria; stojanovpisevski@gmail.com; 2 Research Institute, Research Group “Translational and Computational Neuroscience”, SRIPD, Medical University Plovdiv, 4002 Plovdiv, Bulgaria

## 1. Conceptual History of Psychosis Research

Psychosis research in the contemporary sense of scientific inquiry may be traced as far as the formulation of the “unitary psychosis” concept, or Einheitpsychose, which is usually attributed to Wilhelm Griesinger, Ernst von Zeller, and Heinrich Neumann. It introduced the pre-Kraepeilinian understanding of psychosis as a continuum with multiple manifestations, or forms and stages, rather than as separate nosological categories. However, critically in that view neuropathological “brain dysfunction” was implicated as a mechanism of disease [1].

This doctrine was inherited in 20th century psychopathology by Klaus Conrad, who delineated the difference between unitary endogenous and unitary exogenous psychoses, with the latter being identified with the disturbances of consciousness (orientation), following the intellectual trajectory and earlier contributions of Karl Bonhoeffer.

In Conrad’s perspective, affective disorders and schizophrenia constitute one and the same continuum, with different stages of evolution, many of them resembling mixed clinical states.

The Einheitpsychose concept was opposed by Karl Ludwig Kahlbaum, a precursor of modern nosologism, and this opposition was later developed in the seminal works on psychiatric classification and nomenclature of Emil Kraepelin. In his model of classification, a dichotomy has been introduced between manic depressive psychosis (ciclophrenia) and schizophrenia (dementia praecox in Kraepelinian terms) [2]. He even considered paraphrenia as a separate nosological entity, which was later challenged by St. Stojanov [3]. Simultaneously, Eugen Bleuler coined the term “schizophrenia”, with an emphasis on positive symptoms and their psychodynamic interferences with the deficits in the progression of disease. The modern neo-Kraepelinian doctrine reverberates the crucial significance of the negative symptoms as main vectors of schizophrenia, whereas the neo-Bleulerian view still examines the complex interactions of both positive and negative groups of symptoms in the course of schizophrenia.

On the other hand, the Wernicke–Kleist–Leonhard tradition went even further in an ambitious attempt to construct the most sophisticated classification of systematic and non-systematic schizophrenias, manic-depressive, cycloid psychosis, and atypical psychoses.

In the same period, the school of Andrey Snezhnevsky [4] postulated that schizophrenia has a biologically predetermined longitudinal course with a specific syndrome genesis that he named “pathokinesis”. This concept expanded the frontiers of the diagnostics of psychosis far beyond the anticipated original Kraepelin’s nosology, outlining spectra of the syndrome severity, which ranges from neurotic to organic clinical states. It penetrates into the field of borderline psychopathology, e.g., personality and anxiety disorders, which are defined as possible dimensions of what we may define today as “attenuated psychotic syndrome”. Essentially, Snezhnevsky produced a grading and staging model of psychoses, with high prognostic value and controversial blurred diagnostic values. At the same time, this model was underpinned with a substantial body of biological evidence, converging data from biochemistry, pharmacology, immunology, etc.

All the above developments happened under the umbrella of ICD IX diagnostics. The transitions to ICD X and DSM IV-V have not produced the expected synergy of the applied criteria, measures, and taxonomy. Instead, constant concerns about the validity of psychiatric diagnosis [5] culminated in a crisis of confidence and identity in psychiatric knowledge [6].

This in itself produces the problematic management of psychosis on the levels of early diagnosis, prevention, risk evaluation, and interventions.

In the entire post-Kraepelinian age, another dominant view remained, which was phenomenological psychopathology, named after the publication of the seminal book by Carl T. Jaspers in 1913. This view adopts the “third” way between nosological and anti-nosological traditions. Generally speaking, in this perspective the discrete medical boundaries are abandoned similarly to the unitary psychosis; however, a very comprehensive approach is promoted for the examination of subjective experiences (or phenomena) in terms of signs and symptoms [1].

## 2. Contributions from This Special Issue

This Special Issue summarizes advances in the diagnosis and management of psychosis in the past decade.

It is of particular interest to understand the socio-demographic profile of the emergency psychiatric care users [7] and to deliver structured approaches for the assessment of involuntary treatment [8], because it is involuntary treatment that is supposed to address the most prominent risk behaviors resulting from psychosis, and yet it constitutes one of the most outstanding ethical issues in the management of psychotic disorders. Schizophrenia anxiety is a fundamental and complex condition, differentiated from “neurotic” anxiety, which may be better attuned to social and cultural norms in terms of common sense. Van Staden et al. [9] have designed a novel schizophrenia anxiety rating scale that has the psychometric properties to capture this crucial condition at the intersection between anxiety and psychotic disorders, which is by all means decisive in stipulating a diagnosis and prognosis of disorder.

Critically, the management of psychosis in terms of outcome and general functioning largely depends on the consideration of treatment resistance. In his study, Panov [10] explores the effect of the first drug choice on the development of resistance. There is much evidence to foster the assumption that it is precisely the first antipsychotic drug choice that impacts pharmacodynamics interactions in a similar way to the empirical choice of antibiotic drugs by inducing receptor desensitization.

Neuropeptic-drug-induced Parkinsonism represents another major challenge for the pharmacological treatment of psychosis with conventional anti-psychotics. The investigation of the underlying metabolic patterns by means of positron-emission tomography is the subject of another contribution by Kotomin et al. [11].

Two philosophical papers contemplate the scope of this Special Issue—on epistemic injustice by Wodzinsky and Moskalewicz [12] and on kinesthesia and temporal experience by Sanchez and Moskalewicz [13]. Both focus on different aspects of phenomenological psychopathology and thereby contribute to more qualitative diagnostics of psychotic experiences in a given context.

The problem of movement disorders, as raised in the article by Sanchez and Moskalewicz, is further elaborated in a more quantitative, biologically oriented review by Chepurova et al. [14] on motor imagery and motor execution regarding the application of repetitive transcranial magnetic stimulation.

Finally, Perrottelli et al. [15] deliver a systematic review on the association of EEG measures and cognitive deficits in schizophrenia. As emphasized in the historical overview above, negative symptoms with the prevailing cognitive, affective, and social deficits are considered as the core syndrome of schizophrenia in the classical views of Emil Kraepelin.

Currently, it is precisely the better explanation of the mechanisms, which underpin negative symptoms and the antipsychotic drug resistance, along with the better understanding of the subjective phenomenological experiences of the patient in the relevant social and cultural contexts, which remain among the most exclusive challenges in the field of psychosis research.

The future directions of the field are shaped from the balance and synergy of nomothetic network psychiatry, which contains the potential to produce more valid psychiatric taxonomy on a bio-medical level [16], as well as a values-based comprehensive assessment of the mental disorder in respect to the diverse and often controversial cultural, historical, and social backgrounds of the patients [17].
